# The anorexia of ageing and risk of mortality: More than a story of malnutrition?

**DOI:** 10.1016/j.jnha.2024.100173

**Published:** 2024-02-05

**Authors:** Natalie J Cox, Stephen ER Lim

**Affiliations:** a Academic Geriatric Medicine, Faculty of Medicine, University of Southampton, Tremona Road, Southampton, UK; b NIHR Applied Research Collaboration (ARC) Wessex, University of Southampton, Southampton, UK

Appetite loss or anorexia due to physiological, psychological and socioenvironmental effects of the aging process is termed the anorexia of aging. This syndrome, first described by Morley and Silver in the 1980’s [1], is gaining prominence, with research providing explanations of age-related change to physiological processes driving anorexia, alongside associated social and clinical factors [2,3]. The prevalence of anorexia of aging ranges between 25% and 40%, depending on assessment method and setting, with increased prevalence in hospital and long-term care [2,4]. A relationship between anorexia of aging and mortality was first reported by Cornali et al., in older adults with a recent hospital stay [5]. The link between anorexia of aging and mortality has subsequently been established by a number of longitudinal studies across multiple settings [6], including hospital inpatients [4,7], community dwelling older adults and long-term care residents [[8], [9], [10], [11]].

Despite the association, clear interpretation of the mechanistic relationship between anorexia of aging and mortality, has been limited. There have been observations of correlation between anorexia of aging and frailty and sarcopenia related outcomes [12]. The relationship between anorexia of aging and consequent malnutrition (in the form of protein-energy undernutrition) is better established [6]. Consequently, the link between anorexia of aging and mortality has often been considered through impact on the individuals’ ability to meet protein-energy and nutrient requirements with subsequent malnutrition [12]. Interestingly, Landi et al. observed in community dwellers that while co-existent anorexia and weight loss purveyed an increased risk of mortality, anorexia alone also posed (albeit smaller) risk [9]. This has hinted at a need to better understand the role of malnutrition in the relationship between anorexia of aging and mortality. Dagenais et al’s recent paper ‘Risk of Mortality in Older Adults with Loss of Appetite: An Analysis of Medicare Fee-For-Service Data’ [13], builds upon their previous work [10] with a closer look at the association between anorexia of aging and mortality using data from United States health insurance claims for adults over 65 years old. It provides useful insights into the relationship between anorexia of aging and mortality and raises important questions, which test the view of malnutrition as the main conduit to mortality.

Degenais and colleagues looked at the impacts of Body Mass Index (BMI) category, weight loss and presence of clinically relevant conditions in isolation (including presence of malignancy, heart failure, renal disease) on the relationship between anorexia of aging and mortality [13]. Unsurprisingly, coexistent low BMI or weight loss with anorexia demonstrated a lower rate of survival in stratification. However, the was a clear relationship between anorexia of aging and mortality risk even when stratifying by these factors. In adjusted analyses, the factors which attenuated the relationship between anorexia and mortality to the greatest degree were frailty and comorbidity burden [13]. This preservation of risk of mortality in those with anorexia of aging, separate to the effects of BMI and weight loss, raises questions as to the (likely multiple) mechanisms which underpin this relationship.

Frailty is common in adults with anorexia of aging [12] and has been observed as a medium through which anorexia of aging relates to disability [14]. However, connection with incident frailty is unclear and key to understanding whether anorexia of aging’s role lies in the development of frailty or as a component, also whether it represents symptomology of underlying and undefined processes leading to poor outcomes. Certainly, there is increasing evidence of inflammatory processes being more prevalent in those with anorexia [15], outside of the established cancer cachexia syndrome. Dagenais et al. [13], again helps to pull apart anorexia of aging from the anorexia cachexia syndrome seen in chronic disease and malignancy, as adjustment for these diagnoses did not greatly alter results. As has recently been discussed, taking an approach that considers how age related biological changes and clinical conditions interact is likely to be most fruitful to apprehend mechanisms that underly the relationship between anorexia of aging and mortality [16], of which malnutrition appears only one part.

Studies in ‘healthy’ older adults demonstrating reduction in appetite compared to younger individuals [17], has yielded debate regarding the point at which anorexia of aging might be deemed pathological, hence requiring intervention. This study [13] adds another dimension to this discussion and evidence to the call to increase visibility and focus on anorexia of aging in clinical practice [18], but as a separate phenomenon to established malnutrition risk screening (which is largely dependent on low BMI and presence of weight loss). Also, that appetite loss significant enough to be acknowledged and reported by an older individual should perhaps be explored and acted upon, without reliance on presence of other traditional markers of malnutrition risk. However, what remains to be established is a response to the inevitable ‘what next’ question, as effective management strategies for these anorectic individuals, once identified, are yet to be elucidated [2,19].

## Funding

N.J.C and S.E.R.L receive support from the National Institute for Health Research (NIHR). The views expressed are those of the authors and not necessarily those of the National Health Service, the NIHR or the UK Department of Health and Social Care.

## Conflict of interest

The authors declare no conflict of interest.

## References

[1] Morley J.E., Silver A.J. (1988). Anorexia in the elderly. Neurobiol Aging.

[2] Jadczak A.D., Visvanathan R. (2019). Anorexia of aging - an updated short review. J Nutr Health Aging.

[3] Molfino A., Imbimbo G., Muscaritoli M. (2021). Endocrinological and nutritional implications of anorexia of aging. Endocrines.

[4] Pilgrim A.L., Baylis D., Jameson K.A., Cooper C., Sayer A.A., Robinson S.M. (2016). Measuring appetite with the simplified nutritional appetite questionnaire identifies hospitalised older people at risk of worse health outcomes. J Nutr Health Aging.

[5] Cornali C., Franzoni S., Frisoni G.B., Trabucchi M. (2005). Anorexia as an independent predictor of mortality. J Am Geriatr Soc.

[6] Fielding R.A., Landi F., Smoyer K.E., Tarasenko L., Groarke J. (2023). Association of anorexia/appetite loss with malnutrition and mortality in older populations: a systematic literature review. J Cachexia Sarcopenia Muscle.

[7] Cox N.J., Lim S.E., Howson F., Moyses H., Ibrahim K., Sayer A. (2020). Poor appetite is associated with six month mortality in hospitalised older men and women. J Nutr Health Aging.

[8] Landi F., Lattanzio F., Dell’Aquila G., Eusebi P., Gasperini B., Liperoti R. (2013). Prevalence and potentially reversible factors associated with anorexia among older nursing home residents: results from the ULISSE project. J Am Med Directors Assoc.

[9] Landi F., Liperoti R., Lattanzio F., Russo A., Tosato M., Barillaro C. (2012). Effects of anorexia on mortality among older adults receiving home care: an observational study. J Nutr Health Aging.

[10] Dagenais S., Fielding R.A., Clark S., Cantu C., Prasad S., Groarke J.D. (2023). Anorexia in medicare fee-for-service beneficiaries: a claims-based analysis of epidemiology and mortality. J Nutr Health Aging.

[11] Lin H.Y., Lin Y.C., Chen L.-K., Hsiao F.-Y. (2023). Untangling the complex interplay between social isolation, anorexia, sarcopenia, and mortality: insights from a longitudinal study. J Nutr Health Aging.

[12] Merchant R.A., Woo J., Morley J.E. (2022). Anorexia of ageing: pathway to frailty and sarcopenia. J Nutr Health Aging.

[13] Dagenais S., Clark S., Fielding R.A., Cantu C., Prasad S., Dai F. (2024). Risk of mortality in older adults with loss of appetite: An analysis of Medicare fee-for-service data. J Nutr Health Aging.

[14] Tsutsumimoto K., Doi T., Makizako H., Hotta R., Nakakubo S., Makino K. (2017). The association between anorexia of aging and physical frailty: results from the national center for geriatrics and gerontology’s study of geriatric syndromes. Maturitas.

[15] Pourhassan M., Babel N., Sieske L., Westhoff T.H., Wirth R. (2021). Longitudinal changes of cytokines and appetite in older hospitalized patients. Nutrients [Internet].

[16] de Souto Barreto P. (2022). Poor appetite & aging: the role of physical activity under a geroscience perspective. J Nutr Health Aging.

[17] Giezenaar C., Chapman I., Luscombe-Marsh N., Feinle-Bisset C., Horowitz M. (2016). Ageing Is associated with decreases in appetite and energy intake–a meta-analysis in healthy adults. Nutrients.

[18] de Souto Barreto P., Cesari M., Morley J., Roberts S., Landi F., Cederholm T. (2022). Appetite loss and anorexia of aging in clinical care: an ICFSR task force report. J Frailty Aging.

[19] Ruiz J.G. (2022). Non-pharmacological interventions in anorexia of aging. J Nutr Health Aging.

